# Glucosinolate–Myrosinase Formulations for Adult Obesity: Towards Next Generation of Bioactive Therapies

**DOI:** 10.3390/foods15010013

**Published:** 2025-12-19

**Authors:** Concepción Medrano-Padial, Cassidy Bo Harris, Verónica Mellado-Romero, Raúl Domínguez-Perles, Diego A. Moreno

**Affiliations:** Laboratorio de Fitoquímica y Alimentos Saludables (LabFAS), CSIC, CEBAS, Campus Universitario de Espinardo, 25, 30100 Murcia, Spain; conmedpad@gmail.com (C.M.-P.); cassidybo.harris@um.es (C.B.H.); vmellado@cebas.csic.es (V.M.-R.)

**Keywords:** *Brassica*, glucoraphanin, sulforaphane, adipocyte, lipase, obesity

## Abstract

The rising global prevalence of obesity and metabolic disorders calls for innovative dietary strategies that can modulate key enzymatic pathways involved in lipid and carbohydrate metabolism. This study uncovers the effects of sulforaphane (SFN)-rich broccoli-derived formulations—including liquid and lyophilised forms, as well as two commercial prototypes, Sulforaphan^®^ BASIC and Sulforaphan^®^ SMART, the latter being characterised by the inclusion of an enteric-coated myrosinase enzyme designed to enhance the in situ conversion of glucosinolates (GSL) into bioactive isothiocyanates (ITC)—on lipid and carbohydrate metabolism in 3T3-L1 adipocytes. Across the formulations, total GSL content ranged widely, with GS0 showing the highest levels. Functionally, all SFN-rich formulations significantly reduced intracellular triglyceride content, with the SMART formulation achieving the strongest reduction (11% compared with untreated controls). Across enzymatic assays, we recorded that every formulation inhibited lipoprotein lipase and α-glucosidase activities, with Sulforaphan^®^ BASIC and Sulforaphan^®^ SMART leading to moderate inhibition (40–50%). The potent effect of SMART formulation may be associated with the presence of enteric-coated myrosinase, which enhances the conversion of GSL into bioactive ITC. The gathered evidence provides further insights into the potential of bioactive compounds in cruciferous foods to modulate metabolic health, underscoring their potential role in complementary therapeutic strategies for obesity and its comorbidities.

## 1. Introduction

Nowadays, non-contagious inflammatory chronic illnesses, such as metabolic syndrome, obesity, and diabetes, cause a high mortality rate on a global scale. The growing prevalence of overweight and obesity is worsening the ongoing global health crisis and implies a high cost for the public health systems [1,2]. Obesity is a multifactorial condition, influenced not only by genetic predisposition but also by lifestyle factors (e.g., sedentary lifestyle and inadequate diet), psychological factors (e.g., stress and emotional eating), and socio-environmental factors (e.g., socioeconomic status, cultural habits, and access to healthy foods) [3].

A critical feature of obesity is dysfunctional lipid metabolism, particularly the storage and mobilisation of triglycerides (TG). Moreover, modulating the activity of enzymes such as lipoprotein lipase (LPL) is key to addressing this complex redox–metabolic imbalance [4]. Likewise, carbohydrate digestion and absorption also play a crucial role in such lipid-related metabolic disorders. In this context, α-glucosidase, an enzyme located in the brush border of the small intestine, catalyses the breakdown of disaccharides into absorbable monosaccharides. Hence, inhibiting α-glucosidase can reduce postprandial blood sugar spikes and has become an important therapeutic strategy in managing Type II diabetes (T2D) and metabolic syndrome [5].

Given the multifactorial nature of obesity, its management requires the incorporation of bioactive ingredients as modulators of the referred enzymatic pathways. Indeed, the role of nutrition in modulating metabolic pathways and enzymatic activity has gained attention. Cruciferous vegetables (*Brassica*s), such as broccoli, cauliflower or Brussels sprouts, stand out due to their nutritional profile and bioactive properties. These vegetables contain glucosinolates (GSL) and their cognate bioactive forms, isothiocyanates (ITC), which are found almost exclusively in crucifers [6,7]. The conversion of biologically inactive GSL into their active counterparts occurs through the action of enzyme myrosinase during processing (chopping, cooking, etc.) and mastication. This GSL/ITC conversion is associated with the physicochemical and enzymatic environments during digestion [8,9] and metabolization by the intestinal microbiota [10,11].

Previous research has demonstrated that both the plant part and developmental stage strongly influence the composition of bioactive compounds and their biological activities. For example, broccoli sprouts and florets exhibit distinct profiles of predominant phytochemicals and differential proapoptotic potential [12]. Similarly, studies on kohlrabi sprouts highlight a balance between health-promoting and anti-nutritive compounds, indicating their potential as functional foods [12]. Comparative analyses of broccoli, kale, and cauliflower extracts have also revealed significant antioxidant and antimicrobial activities, reinforcing the functional relevance of *Brassica* species [13]. Furthermore, plant ontogeny strongly affects the antioxidant capacity and bioaccessibility of total phenolic compounds and sulforaphane (SFN), particularly in broccoli sprouts [14].

Among the resulting ITC, SFN has emerged as a promising agent in the complex etiopathogenic mechanisms with disturbed redox/metabolic equilibrium in obesity, metabolic syndrome, and diabetes [4,15]. The benefits of dietary SFN have been shown to significantly reduce obesity-derived chronic inflammation following 10 weeks of broccoli-sprout consumption [16]. Furthermore, studies involving patients with T2D treated with SFN have reported reduced fasting glucose and insulin levels, as well as improvements in insulin resistance indices [17,18]. This cumulative evidence suggests that SFN has the capacity to modulate obesity-associated inflammation and metabolic disorders [19,20], although the magnitude and consistency of these effects require mechanistic clarification.

Nevertheless, gaps remain to be filled in the study of GSL, ITC, and indoles, as the current evidence focuses on their general antioxidant and anti-inflammatory effects, while molecular mechanisms, particularly those involving metabolic enzymes such as LPL and α-glucosidase, remain underexplored. Additionally, most studies emphasise systemic effects or clinical endpoints, overlooking the cellular and enzymatic dynamics that underpin these outcomes.

The present work uncovers the effects of SFN-rich ingredients, derived from byproducts of the frozen food industry, on therapeutic targets associated with obesity. It should be noted that commercial broccoli-derived formulations, including the prototypes evaluated in this study, are complex natural matrices whose GSL profiles and intrinsic myrosinase activity may vary depending on raw material and processing. Therefore, their composition is not comparable to purified GSL standards or isolated myrosinase preparations. In this context, we aimed to evaluate these products in the form intended for use, rather than to standardise all components to identical concentrations. The study evaluated their ability to inhibit LPL and α-glucosidase, as well as their impact on TG metabolism in adipocytes, in vitro, to better understand the potential of these newly developed ingredients as therapeutic candidates for managing obesity and its associated metabolic imbalances. Therefore, the objective of this study is to characterise broccoli-derived formulations with differing GSL and myrosinase contents and to determine their ability to modulate key enzymatic targets linked to lipid and carbohydrate metabolism.

## 2. Materials and Methods

### 2.1. Chemicals and Reagents

Análisis Vínicos S.L. (Tomelloso, Ciudad Real, Spain) and Phytoplan Diehm & Neuberger GmbH (Heidelberg, Germany) provided reagents and standards, respectively. Solvents were purchased from JT Baker (Phillipsburg, NJ, USA). The Millipore water purification system (Bedford, MA, USA) was used to obtain ultrapure water.

The supplies needed to maintain cell culture (trypsin, ethylenediaminetetraacetic acid (EDTA), Dulbecco’s Modified Eagle Medium (DMEM), L-glutamine, foetal bovine serum (FBS), sodium pyruvate, and penicillin/streptomycin) were obtained from Gibco (Thermo Fisher Scientific, Madrid, Spain). The 12-well plates were purchased from Corning (New York, NY, USA).

### 2.2. Tested Formulations

The industrial activity of frozen foods generates byproducts, biomass side-streams or non-commercial parts of vegetables, which remain after separating the marketable products. These materials require a valorisation strategy to support circular economy strategies. Thus, identifying effective methods for their reuse in ingredient preparation and food-grade formulations can be a suitable strategy. Collaborating with Ingredalia SL (Navarra, Spain) was necessary to access the distinct sets of samples that can be obtained in that environment, from initial untreated plant material to pre- and industrial products, as explained in Table 1.

**Table 1:** Formulations rich in glucosinolates (GSL) and myrosinase for evaluation of in vitro anti-obesity potential.

Tested Formulation	Code/Abbreviation
GSL-rich starting material, obtained by processing of broccoli (*Brassica oleracea* L. [Italica Group]) in frozen-food industrial facilities	GS0
GSL Concentrated extract, obtained from GS0, liquid format	GSC
GSC freeze-dried powdered formula	DGSC
Myrosinase-rich concentrated extract, liquid formula	MYRC
Sulforaphan^®^ BASIC encapsulated and powdered formula (including GSC)	BASIC
Sulforaphan^®^ SMART powdered and encapsulated formula (including GSC and MYRC)	SMART

GSL-rich starting plant material was obtained from processing broccoli at industrial facilities (GS0) to prepare a concentrated formula (GSC). This goal was achieved by applying a patented procedure that incorporates excipients (EP 3 123 874 A1). The concentrated GSC was also freeze-dried (DGSC), and the myrosinase-rich concentrated extract (MYRC) used for the preparation of the BASIC and SMART formulations was studied separately. The main interest was to obtain (encapsulated) ingredients rich in GSL/ITC under standardised protocols for further research from in vitro to in vivo and clinical phases, and therefore, “sulforaphane basic^®^” (BASIC) and “sulforaphane smart^®^” (SMART) formulations were considered the prototypes for these purposes and incorporated in this research (Table 1).

The formulations tested in this study correspond to ingredient prototypes developed for nutritional applications, and therefore, they were used in their commercial form. As a consequence, the absolute and relative quantities of individual GSL naturally differ among products, reflecting expected biological variability. The SMART formulation is technologically designed to include an enteric-coated myrosinase enzyme, which is an inherent component of the product and not an independently adjustable variable. For this reason, all formulations were compared as complete matrices rather than standardised to identical GSL or myrosinase concentrations.

### 2.3. Organosulfur Compounds Quantitative Profile (Glucosinolate Analysis)

The previously described formulations (Table 1) were subjected to an intact GSL extraction method [21] where 1 mL of 70% (*v*/*v*) MeOH/deionised water was added to 100 mg of ground sample or 100 µL of extracting solvent and vortexed for homogenization. Subsequently, the sample solutions were placed in a water bath at 70 °C for 20 min, with an intermediate vortex every 5 min, followed by a final ice slurry bath for 5 min to stop the reaction. The sample solutions were then centrifuged at 10,000 RPM for 15 min and filtered using Ø 0.22 µm PVDF filters (Análisis Vínicos, Tomelloso, Spain). After concluding the extraction process, the samples were analysed for GSL content in HPLC-DAD-ESI-MSn (Agilent Technologies HPLC 1200 (Waldbronn, Germany)) coupled to an UltraHCT Ion Trap (Bruker, Bremen, Germany), following previously established protocols available elsewhere [22].

### 2.4. Cell Culture and Differentiation Procedure

The 3T3-L1 cell line (ATCC^®^ CL-173) was obtained from the American Type Culture Collection (Manassas, VA, USA) and maintained at passage numbers lower than 10 in high-glucose DMEM (4.5 g/L) supplemented with 10% (*v*/*v*) foetal bovine serum (FBS), 1% penicillin-streptomycin, 2 mM glutamine, and 2 mM sodium pyruvate. Cells were incubated at 37 °C in a humidified environment with 5% CO_2_ and 95% air. The culture medium was refreshed every 2 to 3 days until the cells reached approximately 80% confluence.

For differentiation purposes, 3T3-L1 preadipocytes were plated in 12-well plates at a density of 2 × 10^4^ cells per well. Once the cells reached confluence, differentiation was induced three days later (designated as day 0) by treating them with differentiation medium consisting of DMEM (4.5 g/L glucose) supplemented with 10% (*v*/*v*) foetal bovine serum (FBS), 1.7 µM insulin, 1.0 µM dexamethasone, 1.0 µM Rosiglitazone, and 0.5 mM IBMX. After 48 h (day 2), the differentiation medium was replaced with a maintenance medium containing 1.0 µM insulin and 10% FBS in DMEM (4.5 g/L glucose). The maintenance medium was refreshed every 48 h until day 10, when the adipocyte differentiation was achieved.

Optical microscopy imaging was performed at selected time points to document the progression of adipocyte differentiation. Images were acquired at days 0, 5, and 10 for the untreated control cells, corresponding to the undifferentiated state, the mid-differentiation stage, and the fully differentiated adipocyte phenotype, respectively. For the treatment groups, microscopy was focused on the day-10 endpoint, as the aim of this determination was to compare the final adipogenic state with the control. Among the tested formulations, the SMART prototype showed the strongest biochemical activity in preliminary assays; therefore, only this formulation was imaged at day 10 to verify the occurrence of the foreseen effects. These images were captured using an optical microscope and included as Supplementary Figure S1.

### 2.5. In Vitro Cytotoxic Test (MTT Assay)

The MTT assay was used to evaluate the cytotoxic effects of the tested items on cell proliferation. Briefly, 3T3-L1 cells were seeded in 96-well plates at a density of 5 × 10^3^ cells/well, and allowed to adhere for 72 h. Subsequently, adipocyte differentiation was induced as described in Section 2.5.

During the differentiation process, cells were treated with the test items at concentrations of 0.001, 0.010, and 0.100 mg/mL. These concentrations were added to both the differentiation and maintenance media at specific time points: days 0, 2, 4, 6, and 8, and maintained for 48 h. Control cells were exposed to unsupplemented culture medium.

On day 10, cell viability was assessed. The media was removed, and MTT solution (1 mg/mL) was added to each well, followed by 4 h incubation at 37 °C. Viable cells reduced the MTT to formazan crystals, which were then dissolved in DMSO. Absorbance was measured at 540 nm using a microplate spectrophotometer. Untreated cells (medium only) were used as a control (considered 100% viable), and results were expressed as a percentage relative to the control.

### 2.6. Quantification of Triglycerides and Assessment of Lipoprotein Lipase and α-Glucosidase Activity

**Figure 1:**
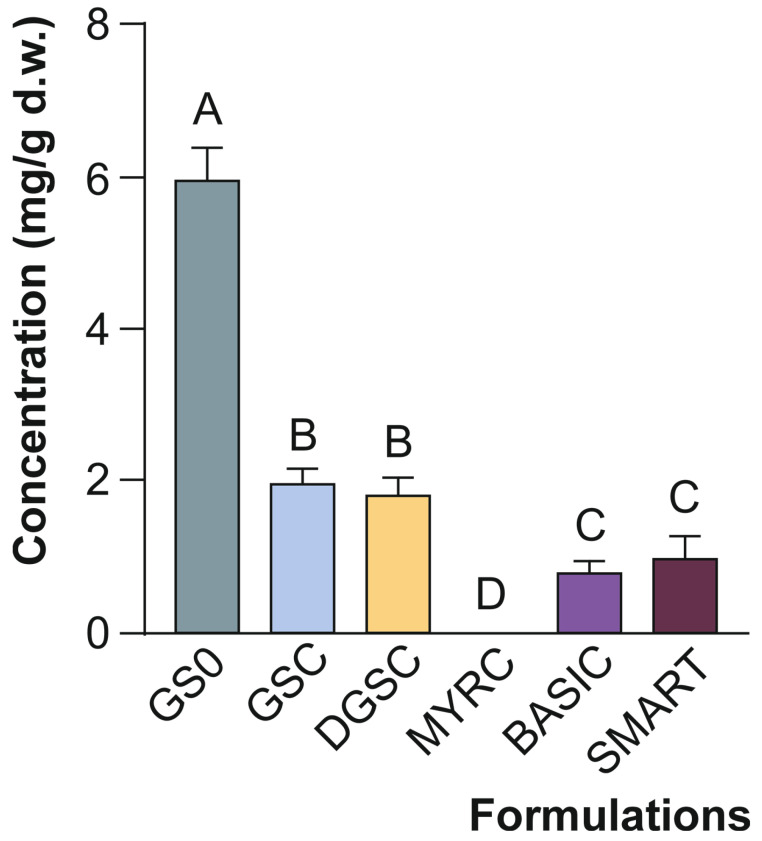
Total glucosinolate contents in the tested formulations. Data represent mean ± SD (*n* = 3). Distinct letters indicate significantly different values at *p* < 0.05 according to one-way analyses of variance (ANOVA) and Tukey’s multiple range test. Refers to Table 1 for abbreviations.

3T3-L1 preadipocytes were treated with 0.1 mg/mL of the selected items (concentration determined based on MTT assay results) by supplementing both the differentiation and maintenance media throughout the differentiation period (on days 0, 2, 4, 6, and 8) for 48 h each time (Figure 1). On day 10, intracellular TG content, as well as α-glucosidase and LPL activities, were assessed using commercial kits following the manufacturer’s instructions (TG: ab65336, and LPL: ab204721, Abcam, Cambridge, MA, USA; and α-glucosidase assay kit MAK123, Sigma-Aldrich, Heidelberg, Germany).

As an internal reference, a pre-differentiation control group was included, consisting of cells maintained in culture medium without adipogenic induction. This group was used to assess basal TG content and enzyme activities before differentiation. Although not included in the final graphical representations to avoid misinterpretation, this control demonstrated the natural increase in the studied parameters upon differentiation and was used as a reference for results interpretation. Comparisons among treatments were made relative to the untreated 3T3-L1 differentiated control cells, which served as the experimental baseline.

### 2.7. Statistical Analysis

All experimental conditions were performed in triplicate (*n* = 3), and the data were expressed as the mean ± standard deviation (SD). According to the normal distribution and homogeneity of variance of the data (determined by the Shapiro–Wilk (<50 samples) and Levene tests, respectively), the obtained results were subjected to one-way analyses of variance (ANOVA), and the statistically significant differences were also evaluated with Tukey’s Multiple Range Test. Significant differences were set at *p* < 0.05. The statistical analyses were performed using SPSS software version 19.0 (SPSS Inc., Chicago, IL, USA).

## 3. Results and Discussion

### 3.1. Characterisation

The characterisation of the formulations listed in Table 1 identified 4 indolic GSL (Glucobrassicin (GB), 4-Methoxy-Glucobrassicin (4-MGB), 4-Hydroxy-Glucobrassicin (4-HGB), and Neo-Glucobrassicin (NGB)), and the aliphatic GSL glucoraphanin (GRA)—parental GSL of SFN. Having identified the specific GSL present in each formulation, we next assessed their overall abundance across products.

The total GSL content is presented in Figure 1, where GS0 represents the optimal processing conditions for the plant material to achieve the highest yield of organosulfur compounds (as a control or lab reference), followed by GSC and DGSC. At the industrial scale, both the GSC and the freeze-dried form of DGSC underwent additional processing steps, which caused a reduction in GSL content. Furthermore, the MYRC does not present any GSL (as expected). Regarding the prototypes of formulations BASIC and SMART, the content in GSL is not significantly different between them.

The ingredient in the BASIC and SMART formulation is incorporated with an excipient, and therefore, the abundance of GSL was reduced accordingly (by 83.9%, on average). The samples processing preserved the intact GSL content, as evidenced by their quantifiable levels in the tested formulations throughout the various steps of the process, from the initial plant material or natural matrix to the final formulated and encapsulated product (Figure 1). After evaluating the total GSL content, we then examined the contribution of each GSL to these profiles.

**Figure 2:**
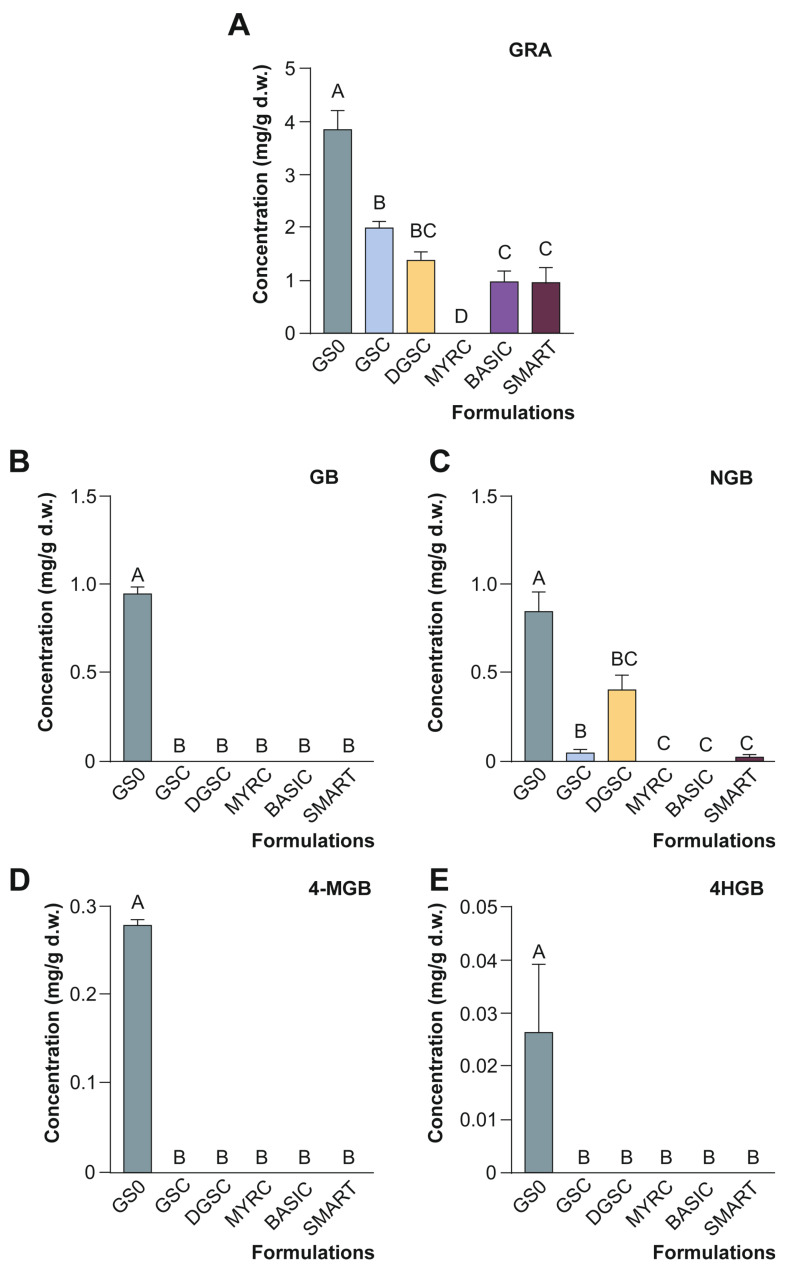
(**A**–**E**) Content of individual glucosinolates across the tested formulations (mg/g d.w.) Data represent the mean ± SD (*n* = 3). Distinct letters indicate significantly different values at *p* < 0.05 according to one-way analyses of variance (ANOVA) and Tukey’s multiple range test. (**A**) GRA, glucoraphanin; (**B**) GB, glucobrassicin; (**C**) 4-MGB, 4-methoxyglucobrassicin; (**D**) 4-HGB, 4-hydroxyglucobrassicin; and (**E**) NGB, neoglucobrassicin. Refers to Table 1 for abbreviations.

The quantification of individual GSL showed that GB, 4-MGB, and 4-HGB were only present in GS0, whereas NGB was also found in GSC and DGSC, albeit in significantly lower amounts (Figure 2). This shift from total to individual GSL levels enables a clearer interpretation of how processing affects specific compounds. When assessing the different formulations on the quantitative GSL profile, GRA, the most abundant GSL in broccoli, which makes up 60% of the total [23], it was found in almost all samples, excluding MYRC. Although freeze-dried, changes in GSL content are expected to be minimal due to the low thermal degradation associated with this process [24]. Consequently, the GRA content of the GS0 formulation was comparable to that observed in fresh broccoli [24] and freeze-dried broccoli [25]. In this regard, the origin of the GSC formulation, an industrial product, may explain this discrepancy since the manipulation, cutting and homogeneity of the drying process under scale-up conditions may result in higher hydrolysis of GSL [26,27]. Consequently, the processing and generation of this product may reduce significantly the original GSL content of the broccoli-based ingredient (Figure 2).

The GS0, without industrial processing, contained a significantly higher GSL content than the additional samples assessed, for both total and individual GSL (Figure 1 and Figure 2). The BASIC and SMART formulations were similar throughout the specific GSL, showing no significant differences at any point in the characterisation process. Moreover, GSC and DGSC variants also presented similar content. However, a closer look at the individual compounds reveals while the level of NGB was higher in DGSC than in the previous step product (GSC), GRA was found in lower concentrations in the former (Figure 2A–E). The freeze-drying (lowering water content) process affected the final content. While freeze-drying can enhance certain compounds [28], it may also lead to losses in others (e.g., thermolabile compounds, volatiles) substances [24]. This duality highlights the importance of selecting the appropriate drying methods based on the target compounds of interest, since specific conditions for the degradation of GSL, depending on processing conditions, are still under discussion [29].

### 3.2. Cell Viability

Cytotoxicity is a key parameter when investigating the biological effects of bioactive compounds, as it allows differentiation between specific cellular modulation and nonspecific effects resulting from decreasing cell viability. This cytotoxic effect is influenced by multiple variables, including the nature of the compound, its concentration, the duration of exposure, and the specific experimental model used [30]. In the context of complex mixtures, such as plant-derived extracts, cytotoxicity evaluation must also consider potential synergistic or antagonistic interactions among their constituents, which may significantly influence their overall toxicity profiles [31].

This study was focused on GSL-rich extracts and myrosinase-containing preparations derived from broccoli. As referred before, the enzymatic activity of myrosinase promotes the hydrolysis of GSL into bioactive ITC, being SFN of remarkable interest in terms of therapeutic properties, but also cytotoxic issues that have been reported at high doses. The molecular mechanisms responsible for the cytotoxic effects of SFN have been attributed to complex and overlapping mechanisms, including the generation of reactive oxygen species (ROS), with a key contribution to triggering apoptosis [32].

**Figure 3:**
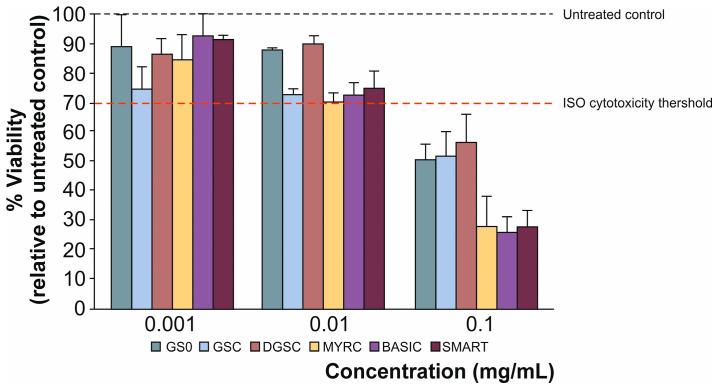
Percentage of viability of 3T3-L1 preadipocytes treated with broccoli-derived formulations at different concentrations (0.001, 0.010, and 0.100 mg/mL), assessed by MTT assay, relative to the untreated control (solid grey line representing 100% viability). A dashed grey line at 70% marks the ISO threshold for cytotoxicity. Data represent the mean ± SD of three independent experiments (*n* = 3). Refers to Table 1 for abbreviations.

In this sense, the assessment of broccoli-derived formulations on the potential cytotoxicity on 3T3-L1 cells during adipocyte differentiation (Figure 3), resorting to MTT assay, allowed identifying a concentration high enough to elicit biological responses without causing excessive cell death.

According to ISO guidelines, a reduction in cell viability below 70.0% compared to untreated controls is considered indicative of cytotoxicity [33]. Based on this criterion, the concentration of 0.010 mg/mL was selected for further experiments, as it consistently maintained viability near or slightly above 70.0% in most of the tested samples (Figure 3). This decision ensures that any observed cellular or metabolic effects can be attributed to the biological activity of the compounds themselves, rather than being secondary to cytotoxicity.

### 3.3. Triglyceride Content

**Figure 4:**
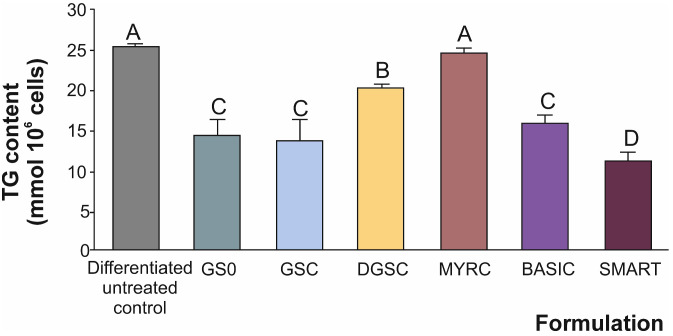
Triglyceride content (TG) (mmol TG × 10^6^ cells) in 3T3-L1 preadipocytes treated with broccoli-derived formulations (0.010 mg/mL). The assay was performed after the adipocytes had fully differentiated. Differentiated untreated refers to differentiated cells that are not exposed to the treatment. Data represent mean ± SD from three independent experiments (*n* = 3). Distinct letters indicate significantly different values at *p* < 0.05 according to one-way analyses of variance (ANOVA) and Tukey’s multiple range test. Refers to Table 1 for abbreviations.

Adipose tissue serves not only as a reservoir for TG storage but also as an active endocrine organ that secretes a variety of adipokines involved in the regulation of energy metabolism, inflammation, and insulin sensitivity [34]. Therefore, quantifying intracellular TG content is key for evaluating the anti-adipogenic potential of bioactive compounds. In this study, treatment with various broccoli-derived formulations at 0.010 mg/mL during 3T3-L1 adipocyte differentiation resulted in significant differences in TG accumulation. Indeed, the differentiation of adipocytes increased the TG content, confirming successful adipogenic induction. In this scenario, among the tested formulations, the SMART one caused the most pronounced reduction in TG content (up to 11.2 mmol × 10^6^ cells), followed by GS0 and the SFN formulations GSC and BASIC (ranging from 13 to 20 mmol × 10^6^ cells, on average) (Figure 4), all demonstrating significant attenuation of lipid accumulation compared to the differentiation control.

Interestingly, DGSC (lyophilised broccoli concentrate) was less effective than the liquid formulation (Figure 4), highlighting a trend observed across assays: liquid formulations consistently performed better than their lyophilised counterparts, possibly due to better preservation of enzymatic activity or compound stability. While lyophilised formulations are generally valued for improved stability and shelf life, previous studies have shown that the processing method can affect the enzymatic activity and phytochemical stability. Such variability may explain why different formulations exhibit differing efficacies in metabolic enzyme inhibition [35]. Indeed, freeze drying is a widely used method for preserving biological materials, including enzymes. However, its effectiveness in maintaining enzymatic activity versus compound stability can vary based on several factors (e.g., drying temperature, rate of freezing, residual moisture content, matrix composition, and presence of stabilising agents) [36]. The enzymatic activity and the stability of the compounds during this process were significantly influenced by the choice of stabilisers and storage conditions, and some specific inhibitors may still be lost during the process [28].

On the other hand, as expected, the cells’ treatment with the myrosinase concentrate (without GSL) did not significantly alter TG content (Figure 4), indicating that the enzyme alone is insufficient to influence lipid metabolism and stressing the specificity of SFN in achieving the biological effect foreseen.

The comparative analysis between the BASIC and SMART formulations demonstrates the relevance of enzymatic activation in modulating lipid metabolism [37]. While both products are derived from GSL-rich materials combined with a biopolymer matrix, the SMART formulation uniquely includes an enteric-coated myrosinase enzyme, designed to preserve enzymatic activity through gastrointestinal transit and promote the in situ conversion of GSL into bioactive ITC, especially concerning the most biologically characterised, SFN.

Optical microscopy observations are presented in Supplementary Materials, Figure S1. Control cells exhibited the expected morphological progression from fibroblast-like preadipocytes at day 0, partial lipid droplet accumulation at day 5, and fully differentiationed adipocytes with abundant intracellular droplets at day 10. In contrast, cells treated with the SMART formulation at day 10 displayed visibly fewer and smaller lipid droplets compared to control day-10 cells. These endpoint images provide morphological support for the biochemical findings, confirming the attenuated adipogenic phenotype induced by the SMART formulation.

### 3.4. Lipoprotein Lipase (LPL) Activity

When testing the range of the broccoli-derived formulations, focusing the present study on LPL activity in differentiating 3T3-L1 adipocytes, as expected, a lower LPL activity in the pre-differentiation control compared to the untreated differentiated control was observed. This finding was consistent with the known upregulation of LPL during adipogenic differentiation [38]. While this group was not included in the bar plots, its activity level helped confirm the adipogenic status of the model and establish the baseline from which inhibitory effects were evaluated. Accordingly, the untreated differentiated control was defined as the basal reference point for inhibitory activity (0%), and all treatment effects were expressed relative to this baseline.

**Figure 5:**
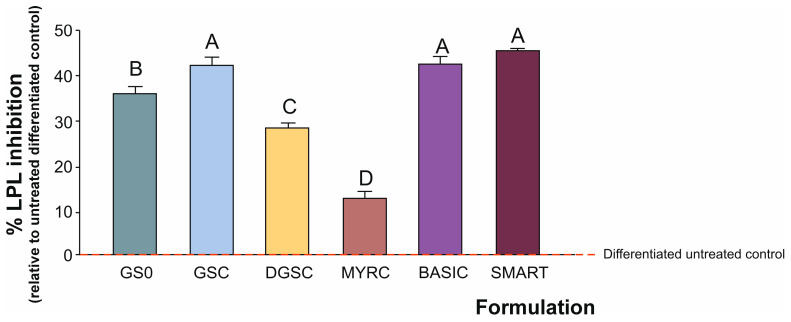
Inhibition of lipoprotein lipase (LPL) activity in 3T3-L1 adipocytes treated with broccoli-derived formulations (0.010 mg/mL). The assay was performed after the adipocytes had fully differentiated. Data are expressed as the percentage of inhibition relative to the untreated differentiated control set as 0% (red line). Data represent mean ± SD from three independent experiments (*n* = 3). Distinct letters indicate significantly different values at *p* < 0.05 according to one-way analyses of variance (ANOVA) and Tukey’s multiple range test. Refers to Table 1 for abbreviations.

The SMART and BASIC formulations were the most effective LPL inhibitors, exhibiting inhibitory effects comparable to those of the concentrated GSC extract (Figure 5). Notably, GSC induced significantly greater inhibition (by 42%) than its source material, GSO (by 36%), which maintained a higher content of GSL and potentially other active constituents of the food matrix (e.g., minerals and phenolic acids). As previously noted, DGSC demonstrated a moderate effect (28% of LPL inhibition) compared to the liquid formulation, likely due to differences in the stability or bioavailability of the bioactive compounds. In contrast, the MYRC showed minimal inhibition, further supporting previous evidence that enzymatic activity alone, in the absence of sufficient substrate, is insufficient to elicit a substantial effect on the molecular targets responsible for the desired functional properties (e.g., concerning LPL inhibition).

These findings suggest that certain broccoli-derived compounds, particularly those containing SFN-parental compounds, can effectively inhibit LPL activity in adipocyte-differentiated 3T3-L1 cells. LPL plays a central role in lipid metabolism by hydrolysing circulating TG, so its inhibition could be associated with anti-adipogenic or lipid-lowering effects [7]. The strongest inhibitory effects were observed with the liquid broccoli concentrate and the SFN-enriched formulations, likely due to a higher availability or more efficient conversion of GSL to ITC (e.g., SFN) [39].

### 3.5. α-Glucosidase

The α-glucosidase is a key enzyme involved in the final steps of carbohydrate digestion by breaking down complex sugars into glucose. Inhibiting this enzyme can delay glucose absorption and is considered an effective way of decreasing postprandial hyperglycaemia [40,41]. These traits offer a relevant strategy in managing postprandial hyperglycaemia and metabolic disorders such as T2D [42].

**Figure 6:**
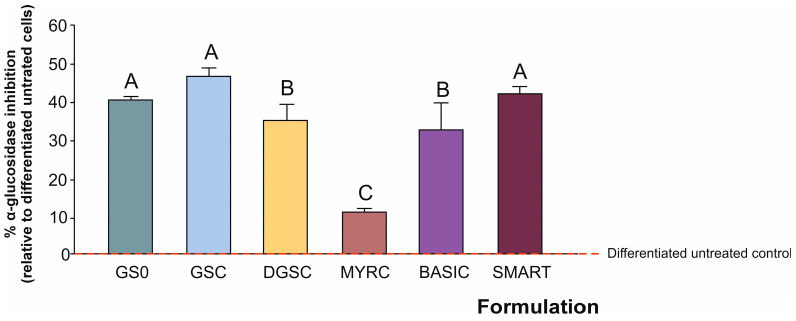
Inhibition of α-glucosidase activity in 3T3-L1 adipocytes treated with broccoli-derived formulations (0.010 mg/mL). The assay was performed after the adipocytes had fully differentiated. Data are expressed as the percentage of inhibition relative to the untreated differentiated control set as 0% (red line). Distinct letters indicate significantly different values at *p* < 0.05 according to one-way analyses of variance (ANOVA) and Tukey’s multiple range test. Differentiation affected the α-glucosidase activity, likely reflecting metabolic adaptations associated with adipocyte maturation. Consistent with previous reports showing that the activity of carbohydrate-metabolising enzymes decreases during the transition from proliferative preadipocytes to lipid-storing mature adipocytes [45,46]. Although the pre-differentiation control was not displayed in the final plot to maintain visual clarity, it served to confirm successful differentiation. Therefore, the untreated differentiated control was used as the reference (0% inhibition) against which all treatment-induced inhibitory effects were calculated. Refers to Table 1 for abbreviations.

In this study, the inhibitory activity of various broccoli-derived formulations against α-glucosidase was evaluated (Figure 6). To contextualise the effects of the treatments, enzymatic activity was also assessed in both undifferentiated and differentiated 3T3-L1 adipocytes.

The α-glucosidase activity was most effectively suppressed by GS0, GSC, and SMART (by 42%, on average). In contrast, BASIC and DGSC produced a similar moderate, yet statistically significant, inhibition (by 33%, on average). As expected, MYRC exhibited the lowest inhibitory activity. The inhibition of the α-glucosidase did not consistently correlate with (nor follow a dose-dependent relationship to) the higher GSL content of the formulations (e.g., GS0). This may be explained by the fact that the concentrated formulations (GSC, SMART, and BASIC) incorporated not only a fraction of GSL with excipients but also other elements of the food matrix as inhibitory coadjuvants. Stronger α-glucosidase inhibition has previously been reported in *Brassica* spp. extracts rich in phenolics [43,44]. The GSL hydrolysis products, as well as other bioactive constituents of the different formulas (e.g., phenolic compounds), likely contributed to the overall α-glucosidase inhibition, although with distinct IC50 values, resulting in a final inhibitory range of 30–40% depending on the treatment. Further comparison with a positive control, as well as studies including bioaccessible fractions of the products, would be valuable to clarify these responses.

The development of standardised GSL-rich ingredients or formulations is of particular interest for dietary interventions and clinical studies. Such products offer several advantages over fresh foods, including consistent dosing despite natural variability. These features are compatible with clinical requirements and allow standardising and coming in easy-to-administer formats (e.g., pills, capsules, or tablets, among others), while retaining most of the attributes of the natural food. Moreover, the main results obtained indicate that it is feasible to develop GSL-rich ingredients such as Sulforaphan^®^-SMART, whose composition and activity profile potentially modulate redox and metabolic imbalances associated with obesity. In particular, these formulations demonstrated significant inhibitory effects on key enzymes involved in lipid and carbohydrate metabolism, which are critical therapeutic targets for obesity and metabolic syndrome, among other pathophysiological conditions.

Despite these advantages, some restrictions have been noticed concerning the scope of most results gathered in the present work. The most relevant limitation is that the formulations assessed are complex broccoli-derived matrices with naturally variable GSL profiles and different myrosinase contents. As a result, the specific contribution of individual GSL to the observed bioactivity cannot be fully attributed when considering a matrix exposed to changing agro-climatic conditions strongly modifying the quantitative phytochemical profile of plant materials. Beyond the starting composition of the material ingested, it is important to consider that human gut microbiota has β-glucuronidase enzymes that can also hydrolyse GSL that escape from the activity of myrosinase released from the plant material in the upper gastrointestinal tract, potentially enhancing the bioavailability and activity of ITC (e.g., SFN) and metabolic derivatives (e.g., SFN-Cys, SFN-NAC, or SFN-GSH, among others) compounds in vivo [47]. Hence, though GSL and ITC have been associated with a high bioaccessibility rate [11], other studies have reported that not all organosulfur compounds supplied by diet and produced upon hydrolysis reactions in the small and large intestine (by vegetable or microbiota-dependent myrosinase) are absorbed and metabolised, as small amounts have been detected in urine and faeces [9]. In this regard, recent research has provided evidence of the myrosinase-like activity exhibited by gut microbiota that can modulate the profile of GSL-derived metabolites, thereby influencing both their systemic availability and biological effects.

Moreover, microbial metabolism of GSL may interact with bile acid signalling pathways, particularly farnesoid X receptor (FXR)-dependent mechanisms, suggesting an additional layer of metabolic regulation that may become relevant in obesity-associated dysbiosis. These interactions represent an emerging area with potential implications for interpreting the in vivo activity of GSL-rich formulations beyond the evidence and scope provided by the present study. Furthermore, to fully understand the effect of the formulations on obesity and overweight, the influence of bile acids should be taken into consideration [48]. The design of the study reflects a practical, application-oriented approach, as the objective was to compare complete ingredient prototypes rather than purified analytical standards.

To overcome these limitations, future research should be developed focused on (i) mechanistic studies with purified GSL types and controlled myrosinase concentrations, (ii) evaluating how gut microbiota metabolism contributes to GSL hydrolysis and formation of derivatives, and (iii) examining SFN-derived metabolites (e.g., SFN-GSH, SFN-Cys, SFN-NAC), which may display distinct biological functions compared with the parent compound.

## 4. Conclusions

In summary, GSL-rich ingredients obtained from industrial processing of Brassica showed superior bioactivity, supporting the notion that formulations retaining the characteristics of the original biological material enhance the performance of bioactive compounds. Therefore, the development of GSL-rich formulations with the biological properties demonstrated will allow their application as nutraceuticals or functional food ingredients with the potential to prevent metabolic imbalances associated with obesity. Beyond their use in controlled dietary interventions, such products are integrated into public health strategies focused on reducing the burden of metabolic syndrome. In this regard, future research should prioritise clinical trials to confirm efficacy and safety in humans, alongside studies addressing the bioavailability, metabolism, and long-term stability of bioactive compounds. In addition, exploring the role of these formulations in other conditions associated with chronic inflammation may broaden their therapeutic potential.

## Data Availability

The original contributions presented in this study are included in the article/Supplementary Material. Further inquiries can be directed to the corresponding authors.

## Supplementary Materials

The following supporting information can be downloaded at: https://www.mdpi.com/article/10.3390/foods15010013/s1. Figure S1: Morphological progression of 3T3-L1 adipocytes during differentiation observed by optical microscopy (100×). Images of untreated control cells were taken at day 0 (undifferentiated) (**A**), day 5 (mid-differentiation) (**B**), and day 10 (fully differentiated) (**C**). Images of cells treated with the SMART formulation were taken at day 10 (**D**), showing reduced lipid droplet accumulation compared to the control.

## Abbreviations

The following abbreviations are used in this manuscript:

4-HGB	4-Hydroxy-Glucobrassicin
4-MGB	4-Methoxy-Glucobrassicin
ANOVA	One-way Analyses of Variance
DMEM	Dulbecco’s Modified Eagle Medium
EDTA	Ethylenediaminetetraacetic Acid
FBS	Foetal Bovine Serum
FXR	Farnesoid X Receptor
GB	Glucobrassicin
GRA	Glucoraphanin
GSL	Glucosinolates
ITC	Isothiocyanates
LPL	Lipoprotein lipase
NGB	Neo-Glucobrassicin
ROS	Reactive Oxygen Species
SD	Standard Deviation
SFN	Sulforaphane
T2D	Type II diabetes
TG	Triglycerides
