# Supplementary material for: Glucosinolate–Myrosinase Formulations for Adult Obesity: Towards Next Generation of Bioactive Therapies
Source: Foods. 2025 Dec 19;15(1):13. doi: 10.3390/foods15010013 (PMC12785495; doi:10.3390/foods15010013)
Supplement: Supplementary file 1 [file foods-15-00013-s001.zip › foods-3974283-supplementary.pdf]

foods

Supplementary Materials

Myrosinase Formulations for Adult Obesity: Toward Next Generation of Bioactive Therapies

Concepción Medrano-Padial, Cassidy Bo Harris, Verónica Mellado-Romero, Raúl Domínguez-Perles * and Diego A. Moreno *

Laboratorio de Fitoquímica y Alimentos Saludables (LabFAS), CSIC, CEBAS, Campus Universitario de Espinardo, 25, 30100 Murcia, Spain; conmedpad@gmail.com (C.M.-P.); cassidybo.harris@um.es (C.B.H.); vmellado@cebas.csic.es (V.M.-R.)

* Correspondence: rdperles@cebas.csic.es (R.D.P.); dmoreno@cebas.csic.es (D.A.M.)

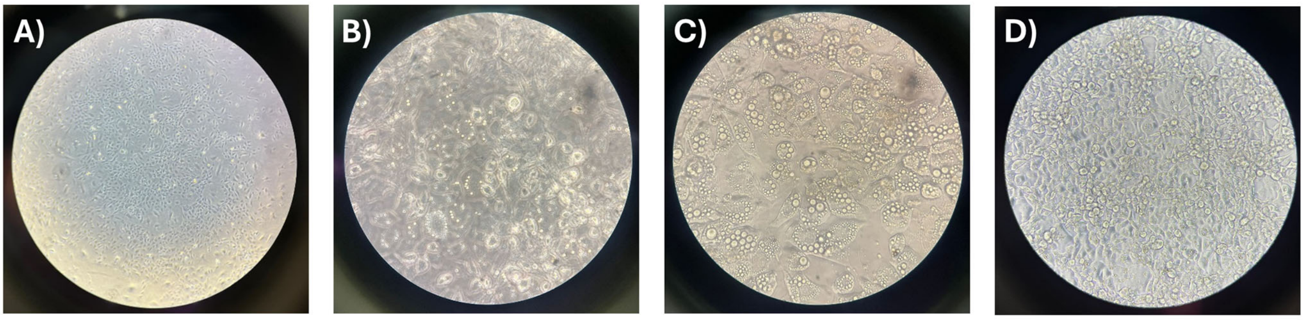

Supplementary Figure S1. *Morphological progression of 3T3-L1 adipocytes during differentiation observed by optical microscopy (100x). Images of untreated control cells were taken at day 0 (undifferentiated) (A), day 5 (mid-differentiation) (B), and day 10 (fully differentiated) (C). Images of cells treated with the SMART formulation were taken at day 10 (D), showing reduced lipid droplet accumulation compared to control.*
